# Hashimoto's thyroiditis attenuates progression of papillary thyroid carcinoma: deciphering immunological links

**DOI:** 10.1016/j.heliyon.2019.e03077

**Published:** 2020-01-08

**Authors:** Oksana Sulaieva, Oleksii Selezniov, Dmytro Shapochka, Nataliia Belemets, Oleksandr Nechay, Yelizaveta Chereshneva, Dibakhan Tsomartova, Marina Ivanova

**Affiliations:** a Laboratory of Pathology CSD Health Care, Kiev, Ukraine; b Ukrainian Research and Practical Centre for Endocrine Surgery, Kiev, Ukraine; c Federal State Autonomous Educational Institution of Higher Education I.M. Sechenov First Moscow State Medical University of the Ministry of Health of the Russian Federation (Sechenov University), Moscow, Russian Federation

**Keywords:** Immunology, Cancer research, Endocrinology, Pathology, Thyroid cancer, Hashimoto's thyroiditis, Tumor immune microenvironment

## Abstract

Although some studies have investigated the clinicopathologic relationships between papillary thyroid carcinoma (PTC) and Hashimoto's thyroiditis (HT), there is still no clear understanding of differences in tumor immune microenvironment for PTC with coexisting HT and HT effect on PTC progression. The aim of this study was to clarify immune-mediated mechanisms of coexisting HT, which might influence PTC progression. 30 patients with histologically confirmed conventional-type PTC and 30 patients with PTC and coexisting HT were enrolled in the study. To analyze the role of immune-mediated links between PTC and HT, immunohistochemical investigation was conducted to count the number of different immune cells including T-cytotoxic cells (CD8), plasma cells (CD138), Treg cells (FOXP3), mast cells (MCT), and M2 macrophages (CD163). It was shown that despite the high number of immune cells in the intact thyroid tissues of PTC patients with coexisting HT there were no significant differences in M2 macrophages, mast cells and Treg counts inside PTC with or without HT. PTC with HT was associated with a higher number of CD8+ cells (P < 0.001) reflecting the ability of immune system to generate and recruit T-cytotoxic cells in tumor area, which can explain the protective effect of HT on PTC progression. Lymph node metastases development was associated with an increased number of mast cells, M2 macrophages and Treg along with a decreased plasma cells count regardless of coexisting HT. However, we did not find significant differences in T-cytotoxic cells quantity in node-positive and node-negative patients with or without HT, which encourages further investigation of immune escape mechanisms in PTC.

## 1 Introduction

Worldwide, an increase in the incidence of papillary thyroid cancer (PTC) has been reported. There are several well-known risk factors facilitating PTC development and progression, with the best-documented environmental cause of PTC being exposure to ionizing radiation. Other risk factors, including sex, obesity, diabetes, smoking, alcohol consumption, dietary nitrates, dietary iodine excess, and genetic factors have also been described (Lloyd et al., 2017). In addition, chronic inflammation can affect thyroid malignant transformation (Oh et al., 2014; Chen and Mellman, 2013; Kim et al., 2009).

A link between cancer and inflammation is well-established but still debatable for inflammatory thyroid diseases and PTC (Galdiero et al., 2016; Paparodis et al., 2014). The most common inflammatory thyroid disease is Hashimoto's thyroiditis (HT), also known as chronic lymphocytic or autoimmune thyroiditis (Kim et al., 2009). The connection between HT and PTC was first described in 1955 (Dailey et al., 1955), and since then numerous epidemiological studies have confirmed high coexistence between HT and PTC ranging from 20% to 85% (Konturek et al., 2013; Lee et al., 2013). In fact, the rate of PTC among patients with HT is several times higher than among those without it (Kim et al., 2009; Lai et al., 2017; Silva de Morais et al., 2019). Although there is confirmed connection between HT and PTC, different studies have not found the link between HT and other thyroid cancers including follicular, medullary, or anaplastic variants (de Paiva et al., 2017).

Apart from its association with the development of PTC, HT has also been suggested to play a protective role against PTC progression (Huang et al., 2011; Zhu et al., 2016). PTC patients with HT appeared to exhibit more favorable clinicopathologic characteristics and a better prognosis than those without HT (Lee et al., 2013; Marotta et al., 2017; Moon et al., 2018). Several studies revealed that PTC patients with coexisting HT exhibited less aggressive clinicopathologic characteristics, as manifested by lower rates of extrathyroidal extension and lymph node metastases (LNM) and showed a longer recurrence-free survival duration (Borowczyk et al., 2019; Graceffa et al., 2019; Konturek et al., 2014; Lun et al., 2013; Moon et al., 2018). Previous studies confirmed a higher rate of microcarcinoma as well as a decrease of LNM rate in patients with coexisting HT (Graceffa et al., 2019; Sulaieva. et al., 2019).

Despite the long story of various studies, the relationship between HT and thyroid malignancies is among the most debatable, with no clear dependencies inferred. When deciphering possible association mechanisms between HТ and PTC, researchers identified different biomarkers which might be involved in neoplastic transformation from HT to PTC. These include RET/PTC rearrangements, p63 protein expression, BRAF mutation, and PI3K/Akt expression (Means. et al., 2019; Xing, 2013; Zeng et al., 2016; Wirtschafter et al., 1997). In addition, immune-mediated mechanisms linking HT and PTC were discovered (Fang et al., 2014; Pyzik et al., 2015). Indeed, malignant transformation in the thyroid gland might be caused by cellular mediators produced by immune cells in chronic inflammation states. The role of CD3+, CD4+ and Th17 cells was also highlighted in several studies (Degertekin et al., 2016; Ward, 2014; Zeng et al., 2018). Still, there is not enough data on the number of cell-mediated and humoral immunity effector cells in patients with PTC and coexisting HT.

On the other hand, malignant cells are closely related to tumor immune microenvironment (TIME) cells that play a critical role in different stages of tumor development and progression by affecting immune surveillance and tumor spread (Baitsch et al., 2012; Binnewies et al., 2018). It is well-known that pro-inflammatory cytokines produced by tumor-associated macrophages and mast cells can promote cancer progression whereas adaptive immunity cells are involved in antitumor effects (Brück, 2016; Caruana et al., 2018). Overall, the complex interplay between infiltrating immune cells and tumor cells has emerged as an essential factor affecting tumor behaviour.

However, there is still no clear understanding of differences in TIME of PTC with or without HT; besides, it remains unclear which immune-mediated mechanisms are involved in HT's effect on PTC progression.

The aim of this study was to clarify immune-mediated mechanisms of coexisting HT impacting PTC progression.

## 2 Materials and methods

This is a retrospective one center-based study that was conducted at Ukrainian Research and Practical Center for Endocrine Surgery. 30 patients with histologically confirmed conventional-type PTC (Group 1) and 30 patients with PTC and coexisting HT (Group 2) were enrolled. Each group included equivalent numbers of patients with and without LNM (Group 1a – patients with PTC and no LNM, n = 15; Group 1b – patients with PTC and LNM, n = 15; Group 2a – patients with PTC, coexisting HT and no LNM, n = 15; Group 2b – patients with PTC, coexisting HT and LNM, n = 15). All the enrolled cases met the following criteria: 1) only females to exclude sex-related differences; 2) 21–54 years of age at the time of hospital admission, 3) histologically confirmed conventional-type PTC sized >1 cm but ≤4 cm (pT1b-2) with no extra thyroid extension. The study excluded patients diagnosed with other malignancies prior to thyroidectomy; patients who received hormonal replacement therapy prior to diagnostics and surgery; patients with Graves’ disease, pregnancy and diabetes mellitus. All patients enrolled into the study provided written informed consent. HT diagnosis was based on clinical and sonographic data, confirmed by serum autoantibodies to thyroglobulin and TPO and by histological slides evaluation. The following criteria for HT diagnostics were used: diffuse lymphocyte infiltration, lymphoid follicles with germinal centers, and Hürthle cell changes. The institutional review board and Local Bioethics Committee of Ukrainian Research and Practical Center for Endocrine Surgery approved the study design and use of human samples.

Patients’ characteristics are provided in Table 1. The age of patients in the 1^st^ and 2^nd^ groups was similar (47.6 ± 0.65 vs 48.5 ± 0.48 years respectively). As it is shown in Table 1, PTC sizes were comparable too (18.1 ± 0.49 (17.1–19.0) and 19.2 ± 0.72 (17.8–20.7) mm respectively.

**Table 1:** Characteristics of patients in groups.

Characteristics	Patients with PTC (n = 30)	Patients with PTC + HT (n = 30)
Age	47.6 ± 0.65 (46.4–48.9)	48.5 ± 0.48 (47.5–49.5)
PTC size	18.1 ± 0.49 (17.1–19.0)	19.2 ± 0.72 (17.80–20.7)
Lymph Node Metastasis		
- Number of patients with LNM	15	15
- Number of patients without LNM	15	15

Data are represented as M±SEM (95% CI).

Tissues processing and evaluation was performed in Laboratory of Pathology CSD Health Care. The tissues were fixed in 10% neutral buffered formalin for 24 h and processed in automatized histoprocessor (Milestone LOGOS Microwave Hybrid Tissue Processor, Milestone, Italy). Paraffin-embedded blocks were cut at 4 μm thickness with the help of Thermo Scientific HM 340E microtome. Histological slides were stained by hematoxylin and eosin using Dako CoverStainer (Agilent, USA). Histopathological assessment was performed with NIKON ECLIPSE E200 microscope (NIKON CORPORATION, Japan).

To analyze the role of immune-mediated links between PTC and HTC, immunohistochemical investigation was conducted to count the number of different immune cells. Immunohistochemical studies were performed according to a standard immunoperoxidase technique. The cells of interest were the following: cytotoxic T-cells (CD8) as effector cells of the cell-mediated antitumor immunity; plasma cells (CD138) as effector cells of the humoral immunity; T regulatory (Treg) cells (FOXP3) involved in self-tolerance mechanisms and immunosuppression; mast cells (MCT); and tumor-associated M2 macrophages (CD163). The list of cell markers, their short descriptions and dilutions are presented in Table 2.

**Table 2:** Characteristics of biomarkers used for immunohistochemical investigation of immune-mediated mechanisms involved in PTC development.

IHC marker	Identified cells	Clone of antibodies manufacturer	Dilution of antibodies
CD8	T-cytotoxic lymphocytes, effector cells of cell mediated immunity	DAKO, clone C8/144B	RTU
CD138	Plasma cells (antibody producing cells), effector cells of humoral immunity	DAKO, clone MI15	1:100
CD163	Macrophages, M2-phenotype	Cell Marque, clone MRQ-26	1:50
MCT	Mast cell tryptase, the marker of the mast cells	Diagnostic Biosystems, clone AA1	1:300
FOXP3	One of the markers of T regulatory cells responsible for immune tolerance and immunosupression	Cell Marque, clone EP340	1:100

When assessing different immune cells, their numbers and spacial distribution were examined. Cells distribution both inside the tumor and in the intact thyroid tissue was considered. Two independent observers performed a blind histomorphometry study at high magnification and assessed all the available fields of vision. The number of immunopositive cells was assessed per 1 mm^2^.

## 3 Statistical analysis

Mathematical and statistical processing of research results was carried out with the help of MedCalc software package. We used Shapiro-Wilka test to check those results for normal distribution and performed further calculations with the help of one-way ANOVA. The results were presented in the form of Mean ± standard error of the mean (SEM) with 95% confidence interval (CI) and considered significant at P ≤ 0.05.

## 4 Results

### 4.1 Immune cells number and distribution in PTC: association with coexisting HT

Assessment of PTC histopathology in patients with coexisting HT revealed high infiltration of both intact thyroid tissues and PTC area. In addition, tumor nodules were surrounded by lymphohistiocytic infiltration. Dense lymphocytic infiltration due to HT was associated with changes in thyroid structure, namely histological architecture alteration and features of cytological atypia.

As expected, the number of all assessed immune cells in intact thyroid tissues was significantly higher in patients from Group 2 (PTC with HT) when compared with PTC alone (Table 3). However, by assessing infiltration inside PTC we did not find significant differences in the number of CD163+ (P = 0.352) and mast cells (P = 0.636) between observed groups. In contrast, the number of CD8+ cells was significantly higher in patients with PTC and HT than in those with PTC alone (169.9 ± 25.2 vs 67.5 ± 12.5 cells per 1 mm^2^; P < 0.001). In addition, CD138+ cells were more numerous in PTC tumors of patients with coexisting HT (P < 0.001).

**Table 3:** Number of immune cells in patients with PTC alone and with coexisting HT.

Immune cells per mm^2^	Patient groups		P
	Group 1 (PTC, n = 30)	Group 2 (PTC with coexisting HT, n = 30)	
M2 macrophages number (CD163)			
Intact thyroid	7.16 ± 0.53 (6.11–8.21)	22.3 ± 2.78 (16.7–27.8)	P < 0.001
In PTC	160.6 ± 13.8 (133.2–187.9)	177.3 ± 8.84 (159.7–194.8)	P = 0.352
Mast cells number (MCT)			
Intact thyroid	2.86 ± 0.41 (2.05–3.68)	12.5 ± 3.22 (5.91–19.0)	P < 0.001
In PTC	35.2 ± 3.64 (27.9–42.4)	32.8 ± 2.47 (27.9–37.7)	P = 0.636
T-cytotoxic cells (CD8)			
Intact thyroid	9.84 ± 0.86 (8.14–11.6)	44.3 ± 8.22 (27.7–60.8)	P < 0.001
In PTC	67.5 ± 12.5 (42.7–92.4)	169.9 ± 25.21 (19.5–220.3)	P < 0.001
Plasma cells number (CD138)			
Intact thyroid	2.22 ± 0.40 (1.41–3.04)	10.0 ± 1.19 (7.30–12.7)	P < 0.001
In PTC	15.9 ± 1.64 (12.6–19.2)	52.9 ± 2.69 (47.4–58.4)	P < 0.001
T regulatory cells number (FOXP3)			
Intact thyroid	0.346 ± 0.11 (0.13–0.56)	5.31 ± 0.74 (3.84–6.77)	P < 0.001
In PTC	2.72 ± 0.42 (1.87–3.56)	5.0 ± 0.75 (3.49–6.51)	P = 0.055

Data are represented as M±SEM (95% CI).

Interestingly, FOXP3+ cells were very rare in the intact thyroid tissue but appeared more often inside PTC tumors in Group 1 patients. In contrast, patients from Group 2 with PTC and HT demonstrated a similar number of FOXP3+ cells in both intact thyroid tissue and PTC areas. When comparing Group 1 and 2 values, we revealed that the number of FOXP3+ cells in intact thyroid tissue was significantly higher in Group 2 patients (P < 0.001). However, there were no statistically significant differences in Treg cells count inside PTC between groups. Anyway, we noticed that FOXP3+ cells were not numerous – up to 5–10 per 1 mm^2^.

**Figure 1:**
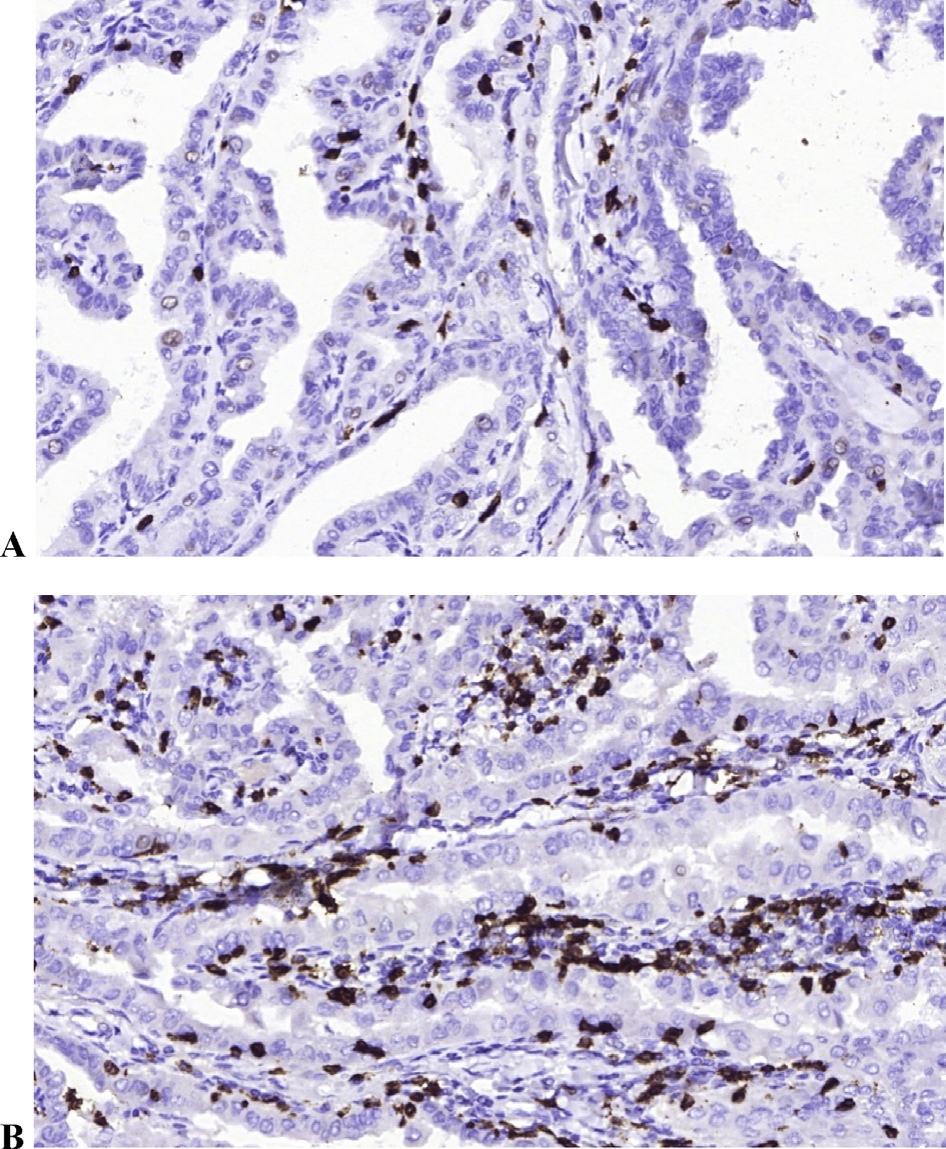
CD8+ cells number in patients with PTC (A) and PTC with coexisting HT (B). HT is associated with dense infiltration with numerous T-cytotoxic cells inside papillae. Immunohistochemistry staining of CD8, ×200.

Therefore, PTC development is associated with a high number of tumor-associated immune cells including plasma cells, T-cytotoxic lymphocytes, M2 macrophages and mast cells. HT was associated with higher Treg cells count in intact thyroid tissue but did not deffer from Treg count in PTC without HT. The only significant difference was the prevalence of CD8+ and CD138 + cells inside PTC tumors coexisting with HT **(**Figure 1**)**.

### 4.2 Immune cells count in PTC tumors: association with regional lymph node metastases

**Figure 2:**
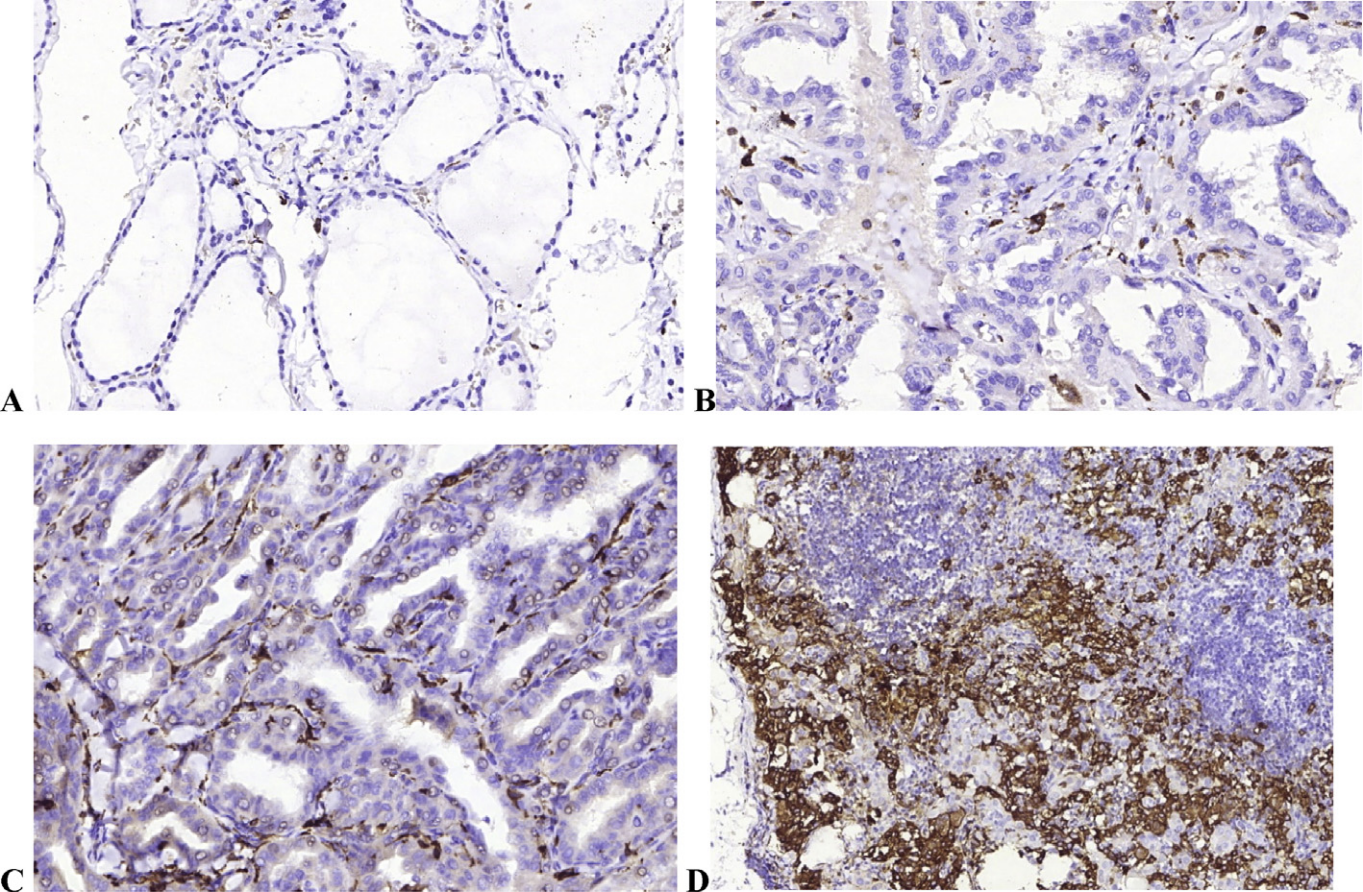
M2 macrophages in intact thyroid tissue (A), PTC without metastases (B), aggressive PTC (C) with metastases in sentinel lymph node (D). PTC metastases in the lymph node are surrounded by numerous M2 macrophages. Immunohistochemistry staining of CD163. Magnification: A and D – ×100; B and C – × 200.

The next step was to compare cell numbers in both groups against the presence of lymph node metastases. When assessing immune cells number in PTC tumors in patients with and without LNM (Table 4), we found out that LNM development was associated with an increased number of myeloid cells (mast cells and M2 macrophages). CD163+ and MCT+ cells count was twice higher in patients with LNM in comparison with the patients with node-negative status in both groups (P < 0,001); however, the difference in M2 macrophages number was much more prominent in patients with PTC without HT. In addition, a strong correlation between CD163+ and MCT+ cells count in tumors and affected lymph nodes was found that allows us to claim the inflammatory mediated signaling pathways activation in PTC metastases development (Figure 2).

**Table 4:** Number of immune cells in patients with PTC in regard with LNM.

Immune cells in PTC	Number of cells (per 1 mm2)		P
	No LNM; n = 30 (Group 1a and Group 2a patients)	With LNM; n = 30 (Group 1b and Group 2b patients)	
M2-macrophages (CD163)	128.1 ± 8.36 (111.6–144.6)	250.6 ± 17.32 (16.1–285.1)	P < 0.001
Mast cells (MCT)	25.9 ± 2.18 (21.5–30.2)	50.9 ± 5.33 (40.3–61.5)	P < 0.001
T-cytotoxic lymphocytes (CD8)	126.2 ± 9.11 (08.4–134.2)	72.1 ± 8.04 (55.9–88.2)	P = 0.149
Plasma cells (CD138)	36.3 ± 3.19 (29.9–42.8)	19.4 ± 3.17 (12.9–25.9)	P = 0.001
T regulatory cells (FOXP3)	2.43 ± 0.43 (1.56–3.29)	6.03 ± 0.63 (4.75–7.31)	P < 0.001

All data are represented as M±SEM (95% CI).

**Figure 3:**
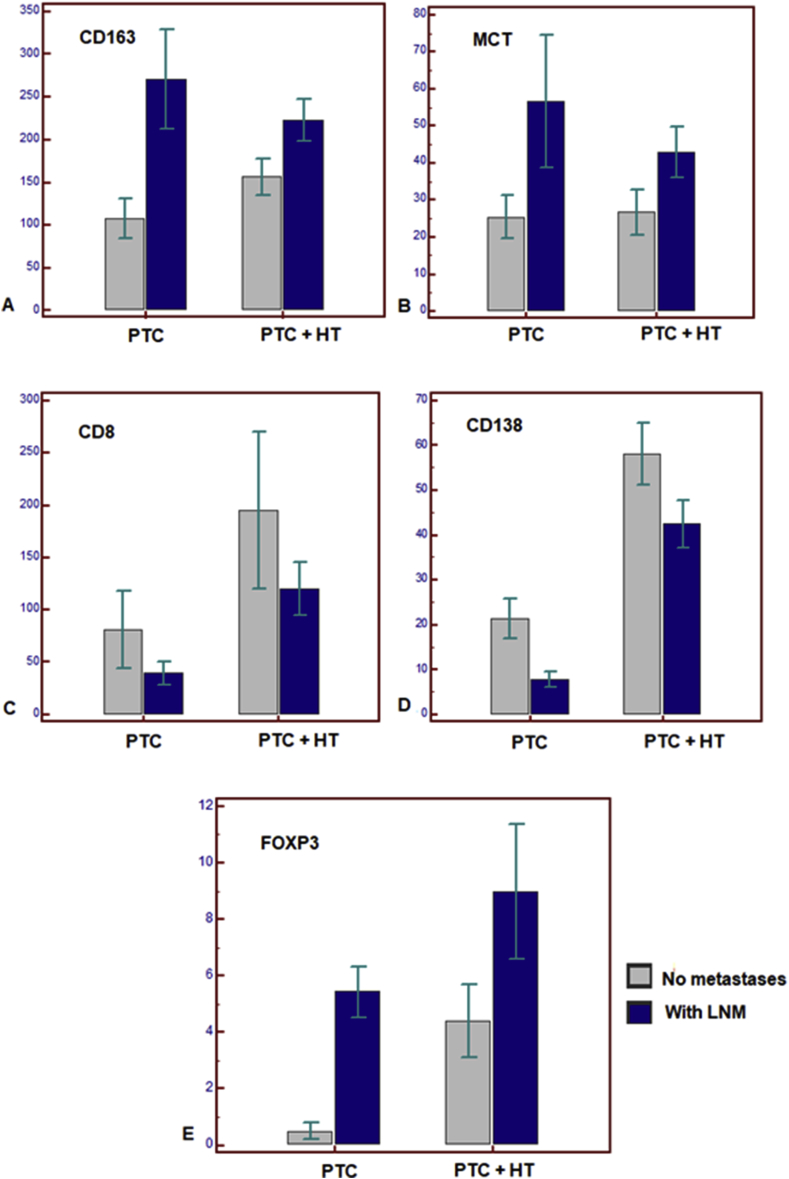
Differences in immune cells number in PTC patients with and without coexisting HT and lymph node metastases (absolute number of cells per mm^2^). Group 1 included patients with PTC (n = 30), 15 of them were node-negative and 15 had LNM. Group 2 included patients with PTC and coexisting HT (n = 30), 15 of them were node-negative and 15 had LNM. All data are represented as M±SD. A – M2 macrophages (CD163), B – mast cells (MCT), C – T-cytotoxic lymphocytes (CD8), D – plasma cells (CD138), E – T-regulatory cells (FOXP3).

When comparing tumor immune microenvironment in PTC with and without LNM, we found significant differences in immune cells number (Table 4). The count of M2 macrophages, mast cells and Treg cells was significantly higher among patients with LNM against node-negative ones. In contrast, the number of plasma cells was lower (P = 0.001) but we did not find significant differences in the amount of T-cytotoxic lymphocytes. To evaluate the impact of HT on immune cells number in terms of LNM, patients were clustered for HT and LNM (Figure 3). In general, independently of coexisting HT, LNM development was associated with a higher number of M2 macrophages, mast cells and T-regulatory cells in combination with the decrease in CD138 + cell count. However, we did not find significant differences in T-cytotoxic cells number in node-positive and node-negative patients regardless of HT. Despite similar changes in tumor immune microenvironment in case of LNM, coexisting HT affected the scale of differences in immunocompetent cells number and their special distribution inside and around PTC tumors in patients with and without metastases. Patients with PTC and no HT demonstrated much more prominent differences in all immune cells counts in tumors with and without LNM, whereas coexisting HT decreased these differences.

## 5 Discussion

As the study shows, PTC development is accompanied by immune cells recruitment and infiltration of tumor area by a high number of innate (M2 macrophages and mast cells) and adaptive immunity cells (plasma cells and T-cytotoxic lymphocytes). Naturally, coexisting HT, which is associated with the increased production of proinflammatory cytokines and chemokines, contributes to different immune cells recruitment (Ward, 2014; Fang et al., 2014; Means et al., 2019) in both non-neoplastic and PTC areas. In patients with coexisting HT we found significant differences in the number of all evaluated immune cells types in non-neoplastic thyroid tissues. However, within PTC nodules different immune cells demonstrated various relationships with coexisting HT. In fact, HT was associated with the increased counts of CD8+ and CD138+ cells within tumor area whereas mast cells and CD163+ cells counts were comparable with those in patients without HT.

Indeed, coexisting HT was associated with high plasma cells number within PTC tumors, which looks natural for autoimmune disease. In addition, the number of plasma cells was higher in patients with PTC without metastases, which can reflect the positive impact of adaptive immunity activation on PTC prognosis. Increased CD138+ cells density has been reported to be related to improved outcome in some cancers including non-small cell lung cancer, ovarian carcinoma, gastric, breast and colorectal cancer (Knief et al., 2016; Kroeger and Milne, 2016; Lohr et al., 2013; Nelson, 2010). So it was only logical for our study to associate the low number of CD138+ cells in PTC tumors with LNM development regardless of coexisting HT.

Previously it was shown that HT is associated with the recruitment of a number of T-lymphocytes to the thyroid gland, including CD4+ cells, cytotoxic T-lymphocytes (CD8+) and Th17 cells (Pyzik et al., 2015). In this study we showed that coexisting HT leads to the increased number of T-cytotoxic lymphocytes (CD8+) both in intact thyroid tissues and within PTC tumors. Tumor-associated lymphocytes have a significant impact on prognosis of different malignancies (Chen and Mellman, 2013; Caruana et al., 2018; Binnewies et al., 2018). It is confirmed that a high number of cytotoxic T-cells contributes positively to anti-tumor effects in different malignancies including breast, colorectal, gastric, cervical and other cancers (Nummer et al., 2014; Maimela, 2019; Thorsson et al., 2018). In fact, the number of CD8+ cells reflects the activation of antitumor response that depends on the cascade of events within tumor immune cycle including tumor antigens recognition, CD8+ cells differentiation with further recruitment of CD8+ T-cells into the tumor site, and tumor cells elimination (Bellone and Calcinotto, 2013; Maimela et al., 2019). These events are influenced by cytokine production and costimulatory signals from antigen-presenting cells - e.g., stimulation of Th1 cells, extracellular matrix and adhesive molecules expression, metabolic, epigenetic and transcriptional factors (Nolz, 2016; Oelkrug and Ramage, 2014). As for our results, increased CD8+ cells number in Group 2 patients shows the impact of coexisting HT on CD8+ cells differentiation and recruitment, which reflects better cell-mediated immunity activation. This could explain the protective effect of HT on PTC growth and progression.

Moreover, based on tumor immune cycle we can postulate that patients with PTC and no HT feature different TIME types compared to those with coexisting HT. Most cases of PTC alone demonstrated a low number of lymphocytes which reflects either a violation of initiation and propagation of antitumor immunity or a lack of T-cells infiltration due to abnormal chemokines or adhesive molecules expression (Chen and Mellman, 2013; Whiteside. 2008). In contrast, PTC with coexisting HT demonstrated high lymphocytic infiltration and CD8+ cells count. Cytotoxic T-cells have different strategies for tumor cells elimination, namely by producing TNF-related apoptosis-inducing ligands or reactive oxygen species and perforin (Anderson K.G et al., 2017). Therefore, tumor growth in case of high CD8+ cells infiltration can be associated with abnormalities of tumor cells recognition and killing related to CD8 disability or immune escape mechanisms activation (Brück et al., 2016; Farhood et al., 2019). For example, tumor cells can express co-inhibitory receptors like programmed death ligand-1 (PD-L1) interacting with programmed death-1 (PD-1) expressed by cytotoxic T-lymphocyte (Maimela et al., 2019; Ulisse. et al., 2019). As a result, such interactions may inhibit tumor cell recognition and elimination by CD8+ cells (Anderson et al., 2017; Hanahan and Coussens, 2012; Maimela et al., 2019).

In addition to effector cells of adaptive immunity, we assessed the number and role of T-regulatory cells. FoxP3+ Treg cells were more frequent within lymphocytic aggregates in patients with PTC + HT when compared with PTC alone. However, there were no significant differences in Treg counts inside PTC and in non-tumor thyroid tissues. The comparison of FoxP3+ cells number in patients with and without LNM reveled the association of high Treg number with PTC invasiveness and progression that could reflect the immunosuppressive effect of Treg cells in PTC. The link between Treg cells and the prognosis was shown in other studies of PTC and other malignancies (French et al., 2010; Hanahan and Coussens, 2012; Cunha et al., 2017). Recent studies revealed that the accumulation of Treg cells in sentinel lymph nodes of breast cancer patients correlates with the presence of metastases within these nodes (Mansfield et al., 2009; Becker and Hald, 2013). In PTC, a high number of Treg cells producing anti-inflammatory factors provides not only a suppressive effect on tumor immune response but also facilitates tumor progression through numerous proangiogenic factors expression (Zou, 2006; Whiteside, 2008). Treg cells inhibit the specialization and function of dendritic cells and their interplay with T-cells, preventing T-cytotoxic cells response and positively contributing to tumor growth (Hanahan and Coussens, 2012). Finally, Treg cells are involved in tumor associated macrophages polarization stimulating M2-macrophages differentiation (De La Cruz-Merino, 2011; Binnewies et al., 2018).

Macrophages can be differentiated by various phenotypes through different activation signals. M1-type macrophages are normally activated by lipopolysaccharide, IFN-γ or tumor necrosis factor (TNF); they secrete pro-inflammatory cytokines and promote inflammation. M2-polarized macrophages are alternatively activated cells (Wang et al., 2015). Their key stimulators are interleukins IL-4, IL-10 and IL-13 produced by Th2-cells and Treg cells under autoimmune inflammation (Martinez et al., 2008; Park et al., 2019). In contrast to M1 phenotype, M2-polarized macrophages are anti-inflammatory; they produce numerous growth factors promoting tumor growth and metastases (Can et al., 2016; Lin et al., 2019). It is interesting to note that tumor-associated macrophages closely resemble M2 macrophages in their function as the former prevent T-cell proliferation and activation by the secretion of restrictive chemokines IL-10, prostaglandins and TGF-β (Wang et al., 2015; Lin et al., 2019). In addition, M2 cells promote tumor invasion by the production of proangiogenic factors including VEGF (Park et al., 2019). In most cases M2 macrophages activities are combined with the stimulation of mast cells secreting numerous chemokines, proangiogenic and growth factors. Our study revealed that PTC tumor immune microenvironment was characterized by a high number of tumor-associated macrophages and mast cells regardless of coexisting HT. Naturally, the increased number of these cells contributed to LNM development. It is worth noting that in addition to elevated CD163+ and MTC+ cells amount within tumors of patients with LNM we found a high number of these cells in affected lymph nodes, which could demonstrate the role of M2 macrophages and mast cells in tumor cells invasiveness and recruitment into lymph nodes.

## 6 Conclusions

PTC with HT is associated with a special immunophenotype featuring a higher number of plasma cells and CD8+ cells inside the tumor that could be related to a higher antitumor response and explain the protective effect of HT on PTC progression. However, there was no statistically significant difference in CD8+ cells count with or without LNM. The prevalence of innate immunity cells with the accumulation of M2 macrophages and mast cells under adaptive immunity cells was associated with more aggressive PTC behavior and LNM development.

## Declarations

### Author contribution statement

Oksana Sulaieva: conceived and designed the experiment; wrote the paper.

Oleksandr Nechay: conceived and designed the experiment.

Nataliia Belemets: performed the experiments; contributed reagents, materials, analysis tools or data.

Oleksii Selezniov: performed the experiments; contributed reagents, materials, analysis tools or data; wrote the paper.

Dmytro Shapochka: analyzed and interpreted the data; wrote the paper.

Yelizaveta Chereshneva: analyzed and interpreted the data; contributed reagents, materials, analysis tools or data.

Dibakhan Tsomartova, Marina Ivanova: analyzed and interpreted the data.

### Funding statement

This research did not receive any specific grant from funding agencies in the public, commercial, or not-for-profit sectors.

### Competing interest statement

The authors declare no conflict of interest.

### Additional information

No additional information is available for this paper.
